# A Study on the Factors Affect the Technology Satisfaction on AI Based Self-service Technology Service Failure in Hotel

**DOI:** 10.1007/978-3-030-65785-7_10

**Published:** 2020-11-28

**Authors:** Lu QianTing, Hee Chung Chung, Namho Chung

**Affiliations:** 1 Department for Informatics, Technical University of Munich, Garching bei München, Bayern Germany; 2 Smart Tourism Education Platform (STEP) College of Hotel and Tourism Management, Kyung Hee University, Seoul, Korea (Republic of); 3 Department of Tourism and Service Management, MODUL University Vienna, Vienna, Wien Austria; 4 Graduate School, Kyung Hee University, Seoul, 02453 Republic of Korea; 5 Smart Tourism Education Platform, Kyung Hee University, Seoul, 02453 Republic of Korea

**Keywords:** Customer satisfaction, AI based self-service technology, Service failure, Service recovery

## Abstract

The advancement of technology following the Fourth Industrial Revolution and the surge in demand for untact services caused by COVID-19 will gradually expand the scope of service automation through artificial intelligence (AI) technologies. In practice, numerous hotels are adopting AI-based service technologies, but it is still in its early stage to provide guidelines for the overall service strategy for this technology. Therefore, this study conducted a study on the failure of AI based self-service technologies (SSTs), service recovery, and the psychological expectations of customers’ SST. An online survey was conducted on respondents who had experience using AI based SSTs, and a total of 370 responses were used for analysis. As a result of structural model analysis through AMOS, it was found that adequate service recovery and low expectations for SSTs were satisfied with SSTs and hotels as a result, even if service failures were experienced. These findings provide specific practical guidelines for many hotels promoting AI-based service automation.

## Introduction

The radical development of information and communication technologies (ICTs) and the Fourth Industrial Revolution have affected almost all industries, and the hotel industry is no exception. Hotels have already adopted self-service technologies (SSTs) such as Kiosk to realize service automation for simple services such as self check-in/check-out. However, the Fourth Industrial Revolution has taken this hotel service automation to the next level, and hotels are providing more sophisticated and diverse self-services based on AI. For example, The FlyZoo Hotel which located in Hangzhou, depending on the Alibaba’s online travel platform, Fliggy, as well as other Alibaba Group business units, such as Alibaba A.I. [1]. As a result, hotel customers are exposed to the hotel’s SSTs and become familiar with the services provided by technology, and many customers are consuming services through technology.

Customers’ demand for service automation is growing even more recently as they experience the global COVID-19 pandemic [10]. Because human-to-human contact can cause the spread of various infectious diseases, hotel customers have come to perceive that contact with hotel service technology is more hygienic [2]. Therefore, more hotels are planning to introduce more diverse technologies and build more diversified and evolved AI-based service automation systems to respond to the post COVID-19 era [10]. However, compared to the rapidly increasing customer demand, AI-based SSTs is in its infancy, both academically and practically. No matter how actively SSTs are used by recent customers, since AI technology has not been applied to the service stage for a long time and unless it is a service failure situation caused by the customers, there may be anxiety about receiving services using the technology [5]. However, when AI-based self-service fails and when service recovery occurs, there has been insufficient discussion on how hotel customers perceive the technology and how they perceive the hotel that introduced the technology. Therefore, a close academic review of AI based services is required. Accordingly, this study aims to provide guidelines for hotels to successfully automate AI based services in response to social phenomena. Specifically, we will look at AI-based self-service failure, and empirically review the factors that cause failure, service recovery, and customer response. In addition, by examining the role of low expectations for SST caused by service failure in service satisfaction, we will additionally examine variables that have not been addressed sufficiently. We expect the results of this analysis to provide effective guidelines for hotels to successfully realize efficient AI-based service automation.

## Theoretical Background and Hypotheses Development

AI based SSTs service failure was theorized as a second-order construct, consist of technology failure, poorly designed interface and excessive customer waiting line which based on previous studies [4, 13]. Service failure leads to two situations. First, when a service failure occurs, the service provider proceeds with a procedure to recover it. At that time, the degree of service recovery varies depending on the degree of service failure, which is generally proportional to the degree of service failure. (H1). Furthermore, it is recognized that the customer cannot easily control the service failure caused by the interaction with AI based technology [6], and as a result, the customer may feel a lot of anxiety [8]. This anxiety eventually leads to low expectations for SST (H2).

*H1–2. AI Based SST Service Failure Has a Significant Effect on Service Recovery and Low Expectation for SST*

Meanwhile, from the viewpoint of customer satisfaction, as has already been demonstrated by numerous studies [e.g., 3], various types of service failures occur in the service delivery process, but service recovery by prompt and appropriate solutions enhances customer satisfaction with service providers [6]. Therefore, even if AI based SSTs cause service failure, a hotel’s adequate service recovery will make customers not only satisfied with AI based SSTs (H3) but also with hotels that introduced and provided this technology (H4).

*H3–4. Recovery from AI Based SST Service Failure is Positively Related to the Satisfaction with AI Based SST and Satisfaction with Hotel.*

Interestingly, low expectations for SST can enhanced satisfaction with SST. Psychologically, satisfaction or happiness depend on individual expectations, not what you feel when something goes well [12]. Therefore, when the customer’s reinforced low expectations for AI-based SST in the negative situation of service failure are converted to a positive situation such as service recovery, it can have a positive effect on satisfaction with SST (H5). It can also have a positive effect on the satisfaction of the hotel that provided it (H6). Lastly, lots of previous studies [e.g., 11] have empirically proved that satisfaction with SSTs has a positive effect on satisfaction with service providers of SSTs (H7).

*H5–6. Low Expectation for SST from AI Based SST Service Failures is Positively Related to the Satisfaction with AI Based SST and Satisfaction with Hotel.*

*H7. Satisfaction with AI Based SST is Positively Related to the Satisfaction with Hotel.*

## Methods

An online survey was conducted. This online survey was conducted from May 2nd, 2020 to May 12th, 2020. Data from a total of 370 respondents who have the experience to use hotel AI based kiosk were used for analysis. Technology failure was measured with 4 items adapted from [13], poorly designed interface was measured with 6 items adapted from [4], and excessive customer waiting line was measured with 5 items adapted from [8]. The scale of service recovery was adapted from [4] including 6 items and the scale of anxiety was adapted from [9] including 4 items. AI based SST satisfaction was measured with 5 items adapted from [14]. Finally, hotel satisfaction was measured with 4 items adapted from [7]. The analysis was been conducted following the steps below. Firstly, coding the data to SPSS format. Secondly, the validity and reliability of all the measurement scales were proved by confirmatory Factor Analysis (CFA) via AMOS was performed to test the measurement model. Finally, Structural Equation Modeling (SEM) analysis and path analysis through AMOS was performed to verify all the seven proposed hypotheses.

## Analysis and Results

**Fig. 1.**
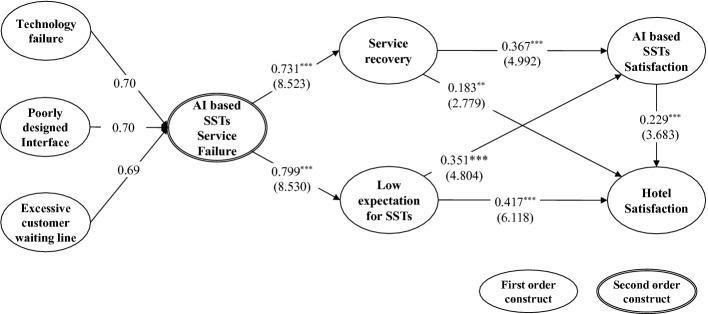
Results of structural model.

To validate our measurement model, this study assessed content, discriminant, and convergent validity using SPSS 23. The content validity was explored and developed from the previous literature. Discriminant validity was tested by comparing the average variance extracted (AVE) associated with each construct (all the AVE 0.6), with the correlations among constructs and the square root of the AVE. Convergent validity was assessed by the composite reliability and Cronbach’s α (all the CR value above 0.6 and all the Cronbach’s α above 0.8). Furthermore, the structural model show a appropriate model fit to the data (χ2 = 583.641; d.f = 289; χ2/d.f = 2.020; CFI = 0.897; NFI = 0.897; GFI = 0.897; AGFI = 0.875; Standardized RMR = 0.071.). As a result of structural model analysis, all hypotheses were supported (see Fig. 1).

Specifically, service failure was found to enhance the service provider’s service recovery behavior and induce low expectations for SST for customers. In addition, enhanced service recovery and low expectations were found to enhance satisfaction with AI-based SST and hotel satisfaction, and AI-based SST also reinforced hotel satisfaction.

## Conclusion

Our findings have the following theoretical and practical implications. In the SST literature, studies on service failure are actively conducted, but studies on low expectation following failure are relatively rare. This study is of academic significance by empirically verifying the effect of low expectation on satisfaction. This interesting role of low expectation deserves attention from hotel practitioners as well. Rather, low expectations for SSTs were found to have a positive effect on customer satisfaction and hotels despite the negative situation of service failure.

Therefore, hotels need to be aware that, rather than emphasizing the ease of self-service and AI service technologies, service recovery such as appropriate assistance in the event of a service failure has more positive results. Despite these implications, this study has limitations in that the study was conducted only in the context of SST. Recently, hotels are introducing various AI-based service agents (e.g., AI-based chatbot, speaker and service robot). Therefore, we intend to expand the research results by conducting research on other types of service agents in the future.
